# First-principles investigation of LaMg_2_Ni and its hydrides

**DOI:** 10.1038/s41598-020-69113-9

**Published:** 2020-07-22

**Authors:** Weiqing Jiang, Yujie Chen, Xiaohua Mo, Xinglang Li

**Affiliations:** 1 School of Physical Science & Technology, Guangxi Key Laboratory for Electrochemical Energy Materials, Guangxi University, Nanning, 530004 China; 2 School of Mathematics and Physics, Key Laboratory for Ionospheric Observation and Simulation, Guangxi University for Nationalities, Nanning, 530006 China

**Keywords:** Energy science and technology, Materials science

## Abstract

Using first-principles density functional theory calculations, the electronic structures of LaMg_2_Ni and its hydrides LaMg_2_NiH_4.5_ (intermediate phase) and LaMg_2_NiH_7_ (fully hydrogenated phase), as well as the H adsorption on LaMg_2_Ni (100) surface were investigated. For comparision, the atomic bonding characteristics of Co- and Pd-doped LaMg_2_Ni, LaMg_2_NiH_4.5_ and LaMg_2_NiH_7_ compounds were also studied. Our aim is to provide new insights into the hydrogenation of LaMg_2_Ni. The results show that the metallic intermediate hydride LaMg_2_NiH_4.5_ with Ni–H covalent bonds may act as the precursor state from the host compound LaMg_2_Ni to the full hydride LaMg_2_NiH_7_. Upon LaMg_2_Ni hydrogenation, the suppression of Mg–Ni and Ni–H interactions as well as the formation of La-H bonds favors for LaMg_2_Ni–H formation.

## Introduction

Hydrogen is an ideal energy carrier, but a major challenge in a future “hydrogen economy” is to develop a safe, efficient and compact hydrogen storage technology. Usually, there are three methods used to store hydrogen, including gas compression, cryogenic liquid storage, and solid state hydrogen storage. Among them, solid state hydrogen storage can offer increased hydrogen density in a safe way^1^.

Magnesium-based alloys are considered to be one promising materials for solid state hydrogen storage due to high storage capacity, abundant resources of magnesium and low cost^2–5^. A typical example is Mg_2_Ni, which can be easily synthesized by combination Mg and Ni, and reacts readily with gaseous hydrogen at moderate temperatures and pressures to form a reversible hydride Mg_2_NiH_4_ containing 3.8 wt% hydrogen^6^. However, the slow absorption/desorption kinetics and the high thermodynamical stability of its hydride severely limit the practical application of Mg_2_Ni^7–10^. It was reported that an improved absorption conditions can be obtained by alloying Mg_2_Ni and rare earth elements to form ternary alloys such as LaMg_2_Ni, as the rare earth hydride (La–H) can effectively catalyze hydriding reactions^8,11–13^. For example, Ouyang et al.^11^ compared pure Mg_2_Ni with LaMg_2_Ni on both thermodynamics and kinetics, and found that LaMg_2_Ni not only has a lower ΔH (− 51 kJ/mol H_2_) and ΔS (− 105 J/K mol H_2_) for hydriding reaction, compared to the ΔH (− 65 kJ/mol H_2_) and ΔS (− 122 J/K mol H_2_) for pure Mg_2_Ni, but also costs less time to reach the saturated hydrogen capacity at lower temperature (1,100 s at 561 K for LaMg_2_Ni vs. 1,800 s at 573 K for Mg_2_Ni^14^). This leads to a decreased hydride stability and an enhanced hydriding kinetics for LaMg_2_Ni, and is ascribed to the presence of LaH_2.46_. Zhao et al.^12^ investigated the hydrogen storage properties of Mg_2_Ni + x wt% LaMg_2_Ni (x = 0, 10, 20, 30) composites, and showed that the existence of LaH_3_ phase contributed to a significant improvement in reversible hydrogen storage properties of the composites over Mg_2_Ni at low temperature. Pei et al.^13^ studied the phase structures and hydrogen storage properties of RMg_2_Ni (R = La, Ce, Pr, Nd) alloys, and reported that the rare earth hydrides (R–H) in the alloys was helpful to improve the thermodynamic properties and accelerate the hydriding kinetics.

In recent years, the ternary compound LaMg_2_Ni which has better hydrogen storage properties than pristine binary compound Mg_2_Ni has attracted considerable attention. Lots of works focus on the hydrogenation/dehydrogenation of LaMg_2_Ni, and verify the facts that the intermetallic compound LaMg_2_Ni absorbs hydrogen reversibly near ambient conditions hereby forming a fully hydrogenated phase LaMg_2_NiH_7_ with LaMg_2_NiH_4.6_/LaMg_2_NiH_4.5_ as intermediate phase; the intermediate phase LaMg_2_NiH_4.6_/LaMg_2_NiH_4.5_ plays an important role in LaMg_2_Ni–H (LaMg_2_Ni hydride) system; the La–H hydrides formed upon hydrogenation shows good catalytic effect on hydriding/dehydriding reactions of LaMg_2_Ni; and the catalysts addition effectively modifies the hydrogen storage performance of LaMg_2_Ni^8,11–13,15–20^. Nevertheless, the hydrogen storage mechanisms of LaMg_2_Ni are still not properly understood. In the present works, the theoretical studies on the electronic structure of LaMg_2_Ni and its hydrides (intermediate phase LaMg_2_NiH_4.5_ and fully hydrogenated phase LaMg_2_NiH_7_) in comparison with corresponding Co- and Pd-containing compounds based on first-principles density functional theory calculations, should be of great interest, since Co and Pd can drastically reduce the reaction time for LaMg_2_Ni-H hydride formation^20^. As in previous studies, Co/Pd was reported to predominantly occupy La/Ni position in LaMg_2_Ni, respectively^20^. Here, Co-containing compounds (LaMg_2_Ni–Co, LaMg_2_NiH_4.5_–Co and LaMg_2_NiH_7_–Co) are introduced by single-substitution of one Co atom at La site in LaMg_2_Ni, LaMg_2_NiH_4.5_ and LaMg_2_NiH_7_, respectively. Similarly, Pd-containing compounds (LaMg_2_Ni–Pd, LaMg_2_NiH_4.5_–Pd and LaMg_2_NiH_7_–Pd) are designed with Pd substitution for Ni. In addition, to further understand the hydrogenation of LaMg_2_Ni, the hydrogen adsorption on LaMg_2_Ni (100) surface is also studied.

## Computational details

Theoretical calculations were carried out using density functional theory (DFT) as implemented in Cambridge Serial Total Energy Package (CASTEP) code^21^. The exchange–correlation function was treated by the generalized gradient approximation of Perdew–Wang 91 (GGA-PW91)^22^. The ultrasoft pseudopotentials with valence states 5*s*^2^5*p*^6^5*d*^1^6*s*^2^ for La, 2*p*^6^3*s*^2^ for Mg, 3*d*^8^4*s*^2^ for Ni and 1*s*^1^ for H were used to describe the core electrons. A plane-wave cutoff energy of 800 eV, and a Monkhorst–Pack k-point mesh of 4 × 3 × 2 for LaMg_2_Ni systems (LaMg_2_Ni, LaMg_2_Ni–Co, LaMg_2_Ni–Pd), 2 × 2 × 2 for LaMg_2_NiH_4.5_ systems (LaMg_2_NiH_4.5_, LaMg_2_NiH_4.5_–Co, LaMg_2_NiH_4.5_–Pd), and 2 × 4 × 2 for LaMg_2_NiH_7_ systems (LaMg_2_NiH_7_, LaMg_2_NiH_7_–Co, LaMg_2_NiH_7_–Pd) were adopted for our calculations. Structural relaxations were carried out by Broyden–Fletcher–Goldfarb–Shanno (BFGS) method^23^ until the residual forces, stresses and displacement were less than 0.03 eV/Å, 0.05 GPa and 0.001 Å, respectively.

**Figure 1:**
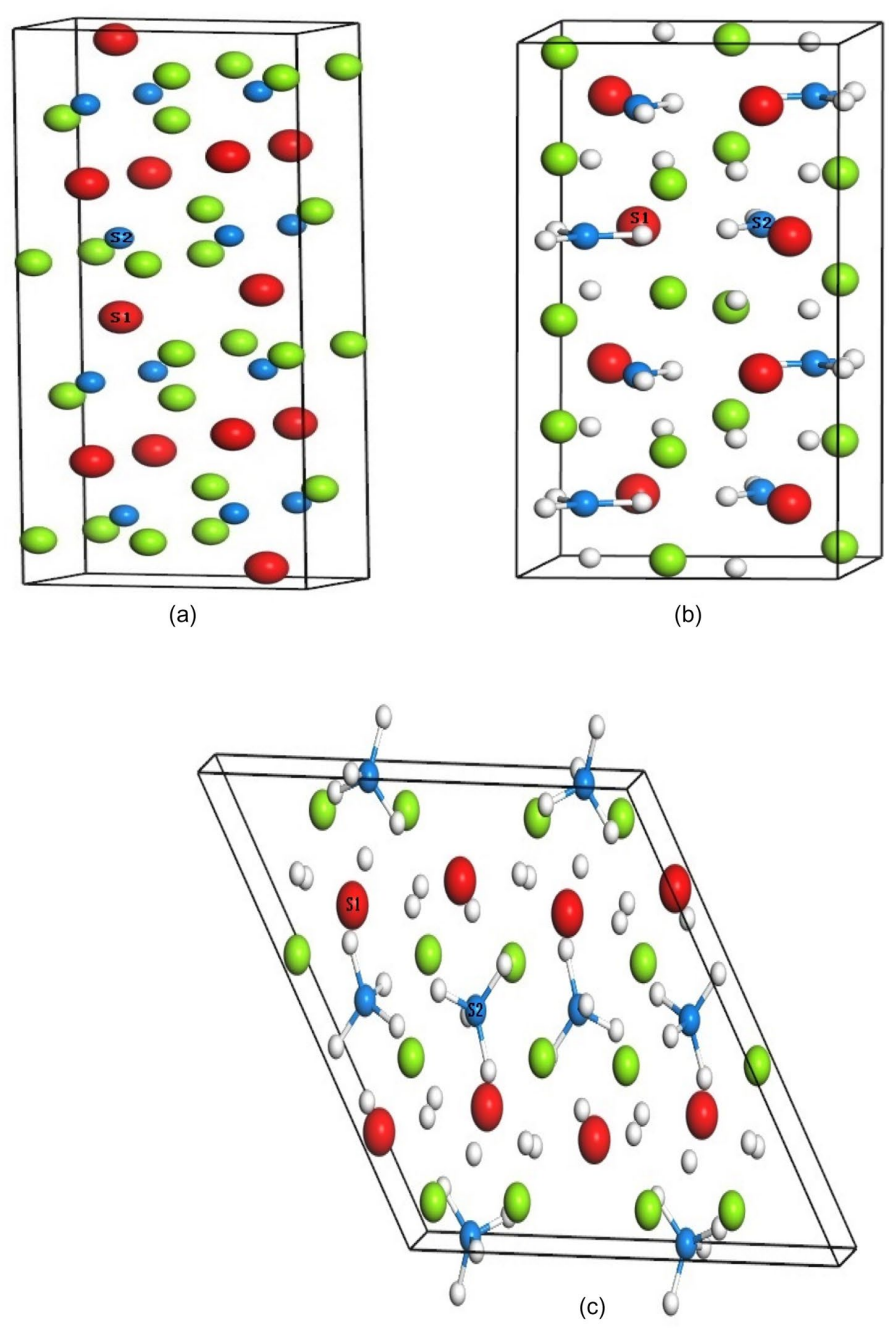
Model of the crystal structure, (**a**) LaMg_2_Ni with 1 × 2 × 1 supercell; (**b**) LaMg_2_NiH_4.5_ with 1 × 2 × 1 supercell; (**c**) LaMg_2_NiH_7_ with primitive cell. Red, green, blue and white spheres denote La, Mg, Ni and H atoms, respectively. S1 and S2 represent substitution sites of La and Ni, respectively.

**Figure 2:**
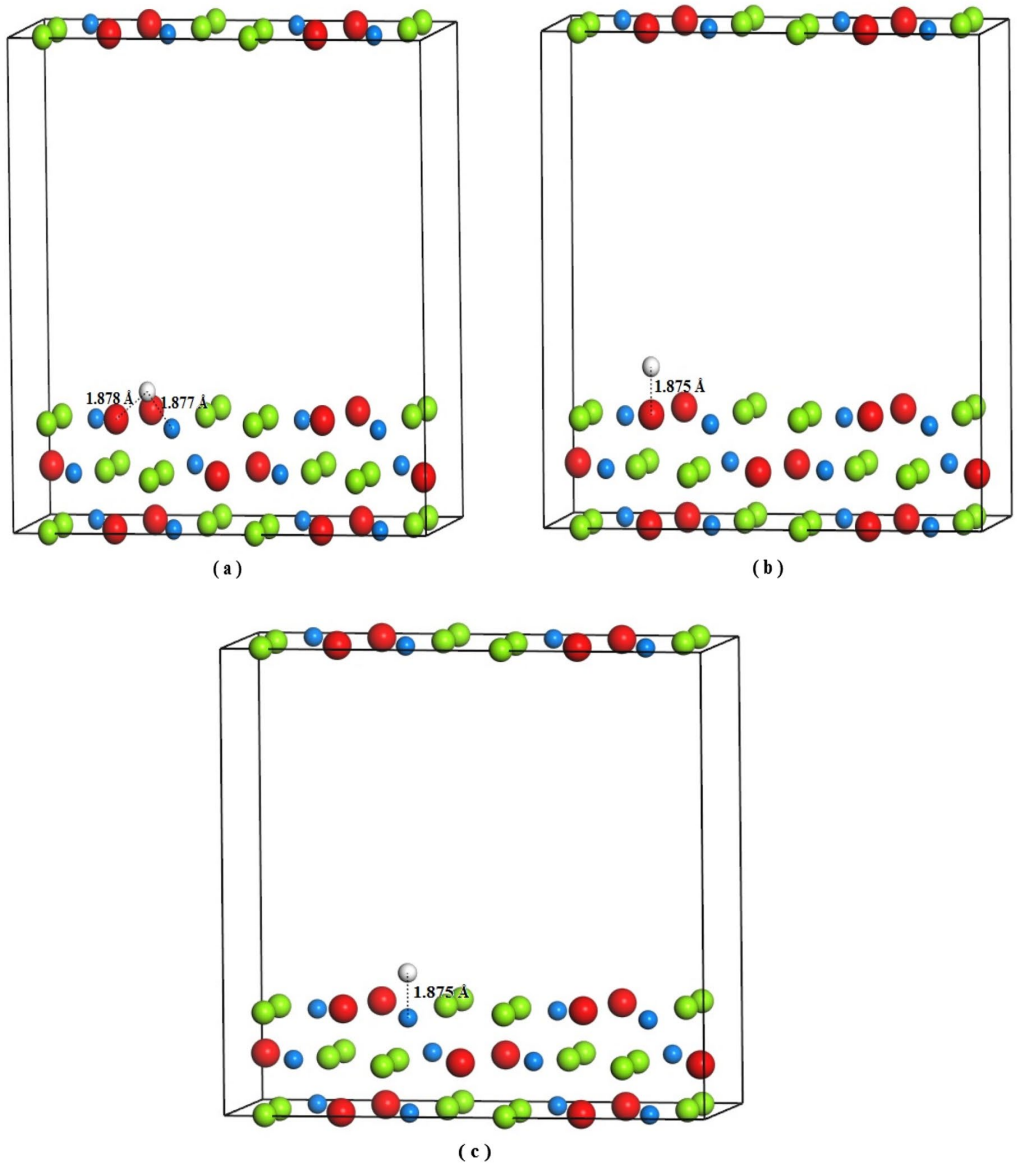
Model of LaMg_2_Ni (100) surface with H adsorbed on the bridge site of La–Ni atoms (**a**), the top site of La atom (**b**) and the top site of Ni atom (**c**). Red, green, blue and white spheres denote La, Mg, Ni and H atoms, respectively. Numbers in the figure are the initial La–H and/or Ni–H distances.

In general, the intermetallic compound LaMg_2_Ni crystallizes in orthorhombic structure with space group Cmcm and lattice parameters a = 4.227 Å, b = 10.303 Å, c = 8.36 Å^15^. Hydrogenation of LaMg_2_Ni at near ambient conditions leads to the formation of intermediate phase LaMg_2_NiH_4.6_/LaMg_2_NiH_4.5_ before the completion of LaMg_2_NiH_7_. Here, the intermediate phase LaMg_2_NiH_4.5_ (space group P21/m and lattice parameters a = 8.602 Å, b = 7.937 Å, c = 6.114 Å, β = 99.53^0^^17^) is selected for our calculations, because the model structure of LaMg_2_NiH_4.5_, which reproduces the powder neutron diffraction (PND) pattern without significant loss of fitting accuracy, can meet with the requirements of computational symmetry, furthermore, the crystallographic parameters of LaMg_2_NiH_4.5_ predicted using DFT calculations are in good agreement with those of LaMg_2_NiH_4.6_ determined from PND experiment^17^. The hydride LaMg_2_NiH_7_ has monoclinic structure with space group P21/c and lattice parameters a = 13.979 Å, b = 4.703 Å, c = 16.025 Å, β = 125.24^0^^15^. Geometry optimizations of lattice constants and atomic positions on bulk LaMg_2_Ni, LaMg_2_NiH_4.5_ and LaMg_2_NiH_7_ gained the relaxed crystals. Calculations for bulk LaMg_2_Ni, LaMg_2_NiH_4.5_ and LaMg_2_NiH_7_ were performed using the relaxed 1 × 2 × 1 (Fig. 1a), 1 × 2 × 1 supercells (Fig. 1b) and primary cell (Fig. 1c), respectively, to ensure all studied systems with the same number of La, Mg and Ni atoms and make the computational results more comparable. In LaMg_2_Ni, LaMg_2_NiH_4.5_ and LaMg_2_NiH_7_ crystals (Fig. 1), partial La (marked with S1) and Ni atoms (marked with S2) are substituted by Co and Pd respectively to introduce Co- and Pd-doped compounds LaMg_2_Ni–Co, LaMg_2_NiH_4.5_–Co, LaMg_2_NiH_7_–Co, LaMg_2_Ni–Pd, LaMg_2_NiH_4.5_–Pd and LaMg_2_NiH_7_–Pd. A structure of LaMg_2_Ni (100) surface was built from the optimized LaMg_2_Ni 1 × 2 × 1 bulk structure (Fig. 1a), which consisted of three La–Mg–Ni layers with 48 atoms (12 La, 24 Mg and 12 Ni). The vacuum space in the surface is 15 Å along the z direction. It is generally believed that H atom can interact with La atom to form La-H hydride upon LaMg_2_Ni hydrogenation^8,11–13^, and Ni atom on La-Ni alloy surface has good catalysis on the surface activity and the initial steps of hydrogen storage (hydrogen adsorption and dissociation)^24–26^. For these facts, here, the initial positions of H on LaMg_2_Ni (100) surface is on the bridge site of La-Ni atoms, the top site of La atom and the top site of Ni atom, as shown in Fig. 2. The initial distances between the adsorbed H atom and the considered La/Ni atom are also described in Fig. 2. During the structural optimization of surface model, all atoms in the top two layers were allowed to relax, whereas the atoms in the bottom one layer were fixed.

## Results and discussion

### Geometry optimization

In our studies, geometry optimizations on bulk LaMg_2_Ni, LaMg_2_NiH_4.5_, LaMg_2_NiH_7_ and their corresponding Co- and Pd-containing compounds (LaMg_2_Ni–Co, LaMg_2_NiH_4.5_–Co, LaMg_2_NiH_7_–Co, LaMg_2_Ni–Pd, LaMg_2_NiH_4.5_–Pd and LaMg_2_NiH_7_–Pd) provided the optimized lattice parameters and cell volume shown in Table 1. As can be seen, the agreement between the optimized results and the available literature values^15,17,20^ is fairly good. This suggests that the present calculations for bulk compounds are highly reliable^27^. Structural relaxations on H-adsorbed LaMg_2_Ni (100) systems results the relaxed La–H and Ni–H distances listed in Table 2. It is found that H adsorption on LaMg_2_Ni (100) surface extends La–H distance, but shortens the Ni–H distance, as compared with their initial distances (Fig. 2). And this extended La–H distance is close to that in LaH_3_ hydride (2.43 Å^16^).

**Table 1:** The optimized lattice constant and cell volume for LaMg_2_Ni, LaMg_2_NiH_4.5_, LaMg_2_NiH_7_ and their corresponding Co- and Pd-containing compounds, in comparison with the available experimental/theoretical data.

Systems	a	b	c	β	V (Å^3^)	References
LaMg_2_Ni	4.426 (4.227)	10.178 (10.303)	8.155 (8.360)	90^0^ (90^0^)	734.83 (728.02)	^15,20^
LaMg_2_NiH_4.5_	8.673 (8.602)	7.982 (7.937)	6.151 (6.114)	99.66^0^ (99.53^0^)	839.566 (823.334)	^17^
LaMg_2_NiH_7_	14.102 (13.979)	4.719 (4.703)	16.155 (16.025)	125.2^0^ (125.24^0^)	878.494 (860.39)	^15^
LaMg_2_Ni–Co	4.207 (4.210)	10.105 (10.278)	8.340 (8.36)	90.0^0^ (…)	733.158 (723.38)	^20^
LaMg_2_Ni–Pd	4.363 (4.24)	10.258 (10.326)	8.259 (8.346)	90.02^0^ (…)	739.291 (730.80)	^20^
LaMg_2_NiH_4.5_–Co	8.889 (…)	15.364 (…)	6.127 (…)	102.398^0^ (…)	817.306 (…)	…
LaMg_2_NiH_4.5_–Pd	8.639 (…)	16.172 (…)	6.154 (…)	99.23^0^ (…)	848.577 (…)	…
LaMg_2_NiH_7_–Co	14.028 (13.959)	4.701 (4.719)	16.151 (16.064)	125.27^0^ (124.89^0^)	869.052 (867.89)	^20^
LaMg_2_NiH_7_–Pd	14.148 (14.003)	4.728 (4.723)	16.177 (16.096)	125.16^0^ (124.79^0^)	884.62 (874.13)	^20^

The numbers outside and inside the bracket correspond to our calculated results and the literature reports.

**Table 2:** The hydrogen adsorption energy (E_ads_, in unit of eV), as well as the bond order (BO), the bond length (BL, in unit of Å) and the scaled bond order (BO^s^, in unit of Å^−1^) between La–H and Ni–H for H-adsorbed LaMg_2_Ni (100) systems.

H-adsorbed LaMg_2_Ni (100) systems	E_ads_	La–H			Ni–H		
		BO	BL	BO^s^	BO	BL	BO^s^
La–Ni bridge site	− 0.565	− 0.05	2.628 (1.878)	− 0.019	0.63	1.668 (1.877)	0.378
Top site of Ni	− 0.311	− 0.10	2.548	− 0.039	0.75	1.577 (1.875)	0.480
Top site of La	− 0.433	0.08	2.437 (1.875)	0.033	…	…	…

Numbers inside the bracket is the initial La–H and/or Ni–H distances.

### Thermal stability

In general, the formation enthalpy ΔH can be used to evaluate the thermal stability of considered compound. A negative formation enthalpy shows an exothermic process. Furthermore, a lower formation enthalpy implies a stronger stability^28^. Here, based on the hydrogenation reaction from LaMg_2_Ni to LaMg_2_NiH_4.5_ (Reaction 1) and LaMg_2_NiH_7_ (Reaction 2), the formation enthalpy for LaMg_2_NiH_4.5_ (ΔH_1_) and LaMg_2_NiH_7_ (ΔH_2_) are calculated by the Eqs. (3) and( 4), respectively.

1 $$ LaMg_{2} Ni + \frac{9}{4}H_{2} \to LaMg{}_{2}NiH_{4.5} $$

2 $$ LaMg_{2} Ni + \frac{7}{2}H_{2} \to LaMg{}_{2}NiH_{7} $$

3 $$ \Delta H_{1} = \frac{4}{9} \times \left[E(LaMg{}_{2}NiH_{4.5} ) - E(LaMg_{2} Ni) - \frac{9}{4}E(H_{2} )\right] $$

4 $$ \Delta H_{2} = \frac{2}{7} \times \left[E(LaMg{}_{2}NiH_{7} ) - E(LaMg_{2} Ni) - \frac{7}{2}E(H_{2} )\right] $$

In Eqs.( 3) and (4), E is the total energy of corresponding systems, which is − 4,169.875, − 4,243.606 and − 4,284.289 eV for LaMg_2_Ni, LaMg_2_NiH_4.5_ and LaMg_2_NiH_7_, respectively. E(H_2_), the energy of hydrogen molecule, is estimated to be − 31.79 eV using a 1,000 Å^3^ cubic unit cell containing two H atoms 0.741 Å apart^29^, and the result agrees well with the literature report of − 31.592 eV^30^. The calculated formation enthalpy for LaMg_2_NiH_4.5_ (ΔH_1_ = − 94.46 kJ/mol H_2_) is found to be lower than that for LaMg_2_NiH_7_ (ΔH_2_ = − 86.84 kJ/mol H_2_), suggesting LaMg_2_NiH_4.5_ may be a thermodynamically stable phase. As in previous studies, LaMg_2_NiH_4.5_ is also expected to be a stable intermediate hydride from the host compound LaMg_2_Ni to the full hydride LaMg_2_NiH_7_^17^.

### Electronic structure

**Figure 3:**
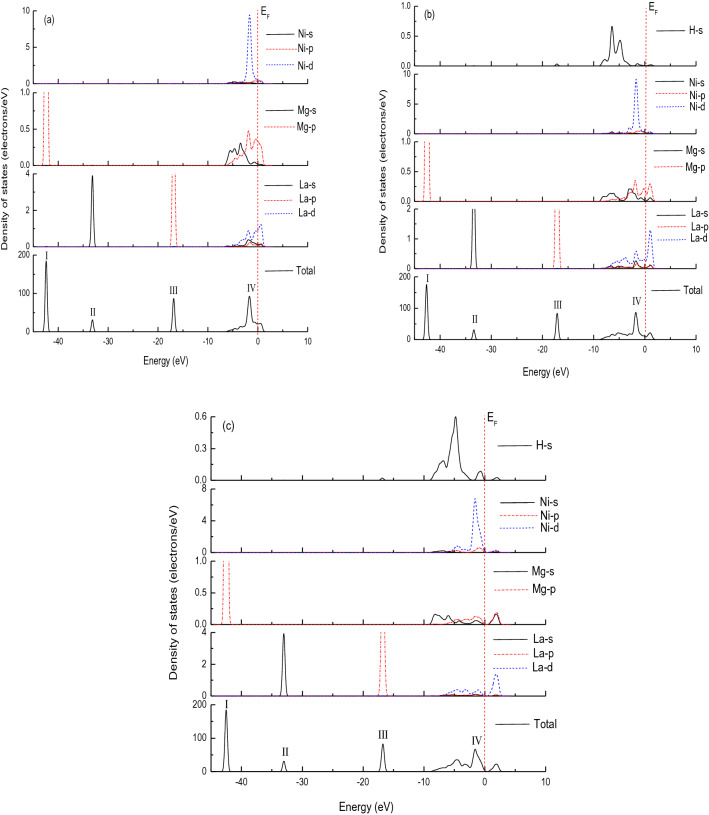
Total and partial density of states for studied compounds, (**a**) LaMg_2_Ni, (**b**) LaMg_2_NiH_4.5_, (**c**) LaMg_2_NiH_7_. The Fermi level is set at zero energy and marked by the vertical dotted line.

To understand the bonding character of bulk LaMg_2_Ni and its hydrides LaMg_2_NiH_4.5_ and LaMg_2_NiH_7_, Fig. 3 shows their total and partial electronic density of states (TDOS and PDOS), where the Fermi level (E_F_) is set at zero energy, and the four main bonding peaks of TDOS are marked with I, II, III and IV, respectively. For the host compound LaMg_2_Ni (Fig. 3a), the peak I has contribution from Mg *p* state. The peak II is dominated by La s state. The peak III is contributed by La *p* state. And the peak IV consists predominantly of Ni d and a few Ni *s*, Ni *p*, Mg *s*, Mg *p*, La *s*, La *p* and La *d* states. When LaMg_2_Ni is hydrogenated to form LaMg_2_NiH_4.5_ and LaMg_2_NiH_7_, the contributions of La, Mg and Ni electronic sates to the peaks I, II, III and IV remain unchanged, except H s state contributes to the peaks III and IV, as illustrated in Fig. 3b and c. Obviously, near the Fermi level (peak IV), the overlap electronic densities originated from La, Mg, Ni, or/and H atomic orbits suggest the atoms La, Mg, Ni, or/and H may interact to each other to form La–Mg, La–Ni, Mg–Ni, or/and La–H, Mg–H, Ni–H bonds. Furthermore, as shown for peak III, La p electrons overlap with H s electrons, also leading to the formation of La–H bond. Referring to the geometrical structure (Fig. 1), however, the distance between La–Mg in LaMg_2_Ni, LaMg_2_NiH_4.5_ and LaMg_2_NiH_7_ (> 3.32 Å), and between La–Ni in LaMg_2_NiH_4.5_ and LaMg_2_NiH_7_ (> 3.09 Å) are so long that La atom is unlikely to interact with Mg and Ni atoms in these systems, thus, La–Mg bond in LaMg_2_Ni, LaMg_2_NiH_4.5_ and LaMg_2_NiH_7_, and La–Ni bond in LaMg_2_NiH_4.5_ and LaMg_2_NiH_7_ can be ignored^31–33^. In addition, considering in peak IV the relatively high value of PDOS at Ni site, the Ni–H bond may have covalent character.

**Figure 4:**
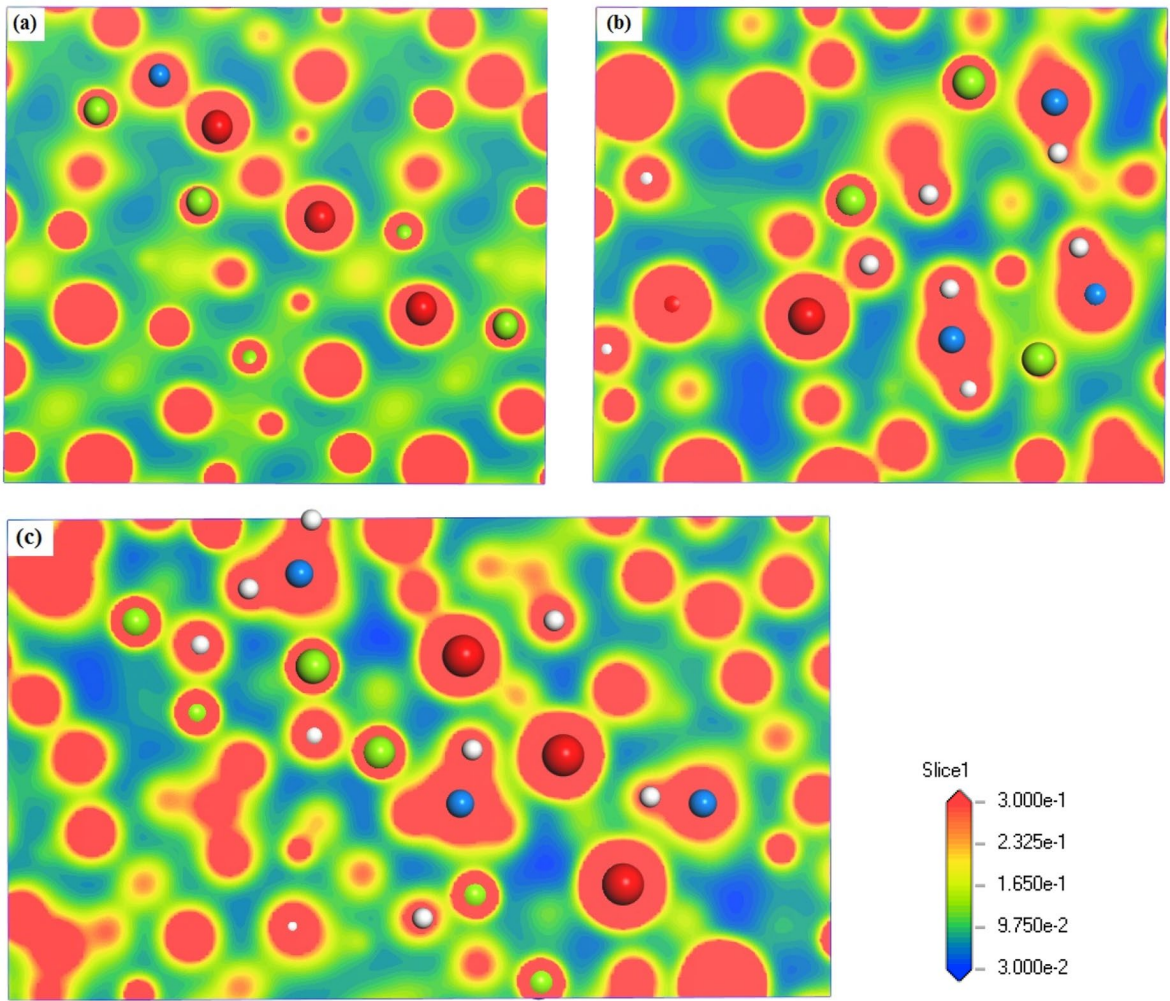
Charge density distribution for studied systems at La, Mg, Ni or/and H sites with the contour lines from 0.03 to 0.3 electrons/Å^3^, (**a**) LaMg_2_Ni, (**b**) LaMg_2_NiH_4.5_, (**c**) LaMg_2_NiH_7_. Red, green, blue and white spheres denote La, Mg, Ni and H atoms, respectively.

The charge density distribution is another intuitive way to investigate the bonding features. Figure 4 shows the results of charge density distribution for bulk LaMg_2_Ni, LaMg_2_NiH_4.5_ and LaMg_2_NiH_7_ at La, Mg, Ni or/and H sites. In this figure, the contour lines are plotted from 0.03 to 0.3 electrons/Å^3^. The shortest distances between La–Mg, La–Ni, Mg–Ni, or/and La–H, Mg–H, Ni–H obtained from Fig. 4 are listed in Table 3. Evidently, in LaMg_2_Ni system, Ni atom can interact with its neighboring Mg and La atoms to form Mg–Ni and La–Ni bonds respectively, as noted from the overlapping electrons between Mg–Ni and La–Ni in Fig. 4a. This formed La–Ni bond, however, tends to be broken from the intake of hydrogen, as the distance between La-Ni increases from 2.901 Å in LaMg_2_Ni to 4.407 Å in LaMg_2_NiH_4.5_ and 3.241 Å in LaMg_2_NiH_7_. The distance between La-Mg reaches to be 3.325 Å in LaMg_2_Ni, 3.700 Å in LaMg_2_NiH_4.5_ and 3.677 Å in LaMg_2_NiH_7_, which is too long to form La-Mg bond. Additionally, as noted in Fig. 4b and c, a directional feature of charge density distribution around [NiH] group contributes to a covalent bond between Ni and H atoms, and this result is consistent with the findings of Miwa et al.^17^. Here, the bonding characteristics among La, Mg, Ni or/and H atoms described in charge density distribution (Fig. 4) are in good agreement with DOS analysis (Fig. 3).

**Table 3:** The shortest distances between La–Mg, La–Ni, Mg–Ni, or/and La–H, Mg–H, Ni–H (in unit of Å) obtained from Charge density plots (Fig. 4) for LaMg_2_Ni, LaMg_2_NiH_4.5_ and LaMg_2_NiH_7_.

Systems	La–Mg	La–Ni	Mg–Ni	La–H	Mg–H	Ni–H
LaMg_2_Ni	3.325	2.901	2.733	…	…	…
LaMg_2_NiH_4.5_	3.700	4.407	2.670	2.377	2.008	1.560
LaMg_2_NiH_7_	3.677	3.241	2.682	2.641	1.954	1.565

To elucidate the bonding characteristics quantitatively, Mulliken population analysis is applied to bulk LaMg_2_Ni, LaMg_2_NiH_4.5_ and LaMg_2_NiH_7_, including the average bond order (BO), average bond length (BL) and scaled bond order (BO^s^), and the results are shown in Table 4. Here, BO^s^, the average bond order (BO) per unit bond length (BL), is estimated using the formula BO^s^ = BO/BL, and can be used to evaluate the relative bonding strength between atoms^31,32,34–36^. A bond with positive BO^s^ is expected to be a covalent nature. Moreover, the higher the BO^s^ is, the stronger the bonding interaction is. The absent La–Ni bonds in LaMg_2_NiH_4.5_ and LaMg_2_NiH_7_, and La–Mg bonds in LaMg_2_Ni, LaMg_2_NiH_4.5_ and LaMg_2_NiH_7_ (not shown in Table 4), and the presence of Mg–Ni, Ni–H, La–H and Mg-H bonds in studied systems are consistent with those described in Figs. 3 and 4. Evidently, with hydrogen absorption, the atomic interaction between Mg-Ni becomes weaker, but between Ni–H becomes stronger, as noted from the decreased scaled bond order between Mg–Ni (BO^s^_Mg–Ni_) and the increased scaled bond order between Ni–H (BO^s^_Ni–H_) in Table 4. As in previous study, the Mg–Ni and Ni–H interactions are reported to affect directly the phase stability of binary Mg_2_Ni intermetallic hydride based on the electronic structures of Mg_2_Ni intermetallic hydride containing a variety of alloying elements^37^. In the present study, the impact of Mg–Ni and Ni–H interactions on the hydrogenation of ternary compound LaMg_2_Ni (the product of La and Mg_2_Ni by ball milling) is discussed based on Mulliken population analysis of bulk LaMg_2_Ni and its hydrides LaMg_2_NiH_4.5_ and LaMg_2_NiH_7_ in comparison with corresponding Co- and Pd-containing compounds (LaMg_2_Ni–Co, LaMg_2_NiH_4.5_–Co, LaMg_2_NiH_7_–Co, LaMg_2_Ni–Pd, LaMg_2_NiH_4.5_–Pd and LaMg_2_NiH_7_–Pd, Table 4), because Co and Pd addition can drastically reduce the reaction time for LaMg_2_Ni–H hydride formation^20^. It is found in Table 4 that that Pd, especially Co addition weakens the Mg-Ni interaction, as compared to corresponding Co- and Pd-free compounds. Moreover, with hydrogen uptake, the Mg–Ni interaction for Co- and Pd-containing compounds also gradually decreases, as the BO^s^_Mg–Ni_ is eventually reduced by 74.6% (LaMg_2_Ni–Co system) and 72.9% (LaMg_2_Ni–Pd system). We believe that the decreased Mg–Ni interactions are beneficial for the improvement of hydrogenation properties of LaMg_2_Ni. Referring to the Ni–H bonds, it is formed as the hydrogenation reaction to the intermediate hydride phase, and becomes stronger to the full hydride phase, showing a covalent nature with the bond order between Ni–H being positive (BO_Ni–H_ > 0)^31,32,34,38^. Miwa et al.^17^ and Sato et al.^19^ had proposed that the intermediate hydride phase LaMg_2_NiH_4.5_ may play as precursor state for the following complex hydride LaMg_2_NiH_7_ formation as the Ni–H bonds in LaMg_2_NiH_4.5_ are essentially covalent nature similar to those in LaMg_2_NiH_7_, which subsequently provides the reduction of energy barrier for LaMg_2_Ni hydrogenation. According to this viewpoint, our intermediate hydrides LaMg_2_NiH_4.5_, LaMg_2_NiH_4.5_–Co and LaMg_2_NiH_4.5_–Pd with Ni–H covalent bonds may also act as precursor states for the following hydrogenation reaction to LaMg_2_NiH_7_, LaMg_2_NiH_7_–Co and LaMg_2_NiH_7_–Pd, respectively. In addition, it is worth noting that the introduction of Co and Pd not only hits growth in BO^s^_Ni-H_ from 12.2% (LaMg_2_Ni system) to 3.95% (LaMg_2_Ni–Co system) and 11.43% (LaMg_2_Ni–Pd system) even in the case of increasing Ni–H interaction with more hydrogen absorption, but also weakens the Ni–H interactions at fully hydrogenated states, as the BO^s^_Ni–H_ of 0.447 Å^−1^ for LaMg_2_NiH_7_–Co and 0.468 Å^−1^ for LaMg_2_NiH_7_–Pd are lower than that of 0.469 Å^−1^ for LaMg_2_NiH_7_. Interestingly, both the growth of BO^s^_Ni–H_ from intermediate state to fully hydrogenated state and the BO^s^_Ni–H_ at fully hydrogenated states decrease in the order of LaMg_2_Ni system (12.2%, 0.469 Å^−1^) > Pd-containing LaMg_2_Ni system (11.43%, 0.468 Å^−1^) > Co-containing LaMg_2_Ni system (3.95%, 0.447 Å^−1^). This descending order is just consistent with the reaction time for LaMg_2_Ni–H formation, LaMg_2_Ni systems (7.5 h) > Pd-containing LaMg_2_Ni systems (3 h) > Co-containing LaMg_2_Ni systems (1.5 h)^20^. Thus, it is reasonable to conclude that the suppression of Ni–H interaction upon LaMg_2_Ni hydrogenation should accelerate LaMg_2_Ni–H formation, and subsequently improve the hydrogenation performance of LaMg_2_Ni. A similar example is found on Mg_2_Ni system. Cu doping can accelerate Mg_2_Ni hydride reaction followed with a reduction in Ni–H interaction in Mg_2_NiH_4_ hydride^39,40^. In the case of the La–H bonds formed upon hydrogenation of LaMg_2_Ni, the La-H bond length (BL_La–H_) in intermediate hydride LaMg_2_NiH_4.5_ (2.475 Å, Table 4) is very close to that in binary hydride LaH_3_ (2.43 Å^16^). This characteristic is also embodied in Co- and Pd-containing LaMg_2_Ni systems with the BL_La–H_ of 2.506 Å in LaMg_2_NiH_4.5_–Co and 2.475 Å in LaMg_2_NiH_4.5_–Pd (Table 4). Pei et al.^8^ had investigated the effect of La hydride compound on hydriding process of LaMg_2_Ni, and showed that LaMg_2_Ni would decompose to LaH_3_ during hydrogenation, and this La hydride compound was helpful to improve the hydrogen storage property of LaMg_2_Ni at low temperature. Many previous studies also verified that the La–H hydride could show good catalytic effect on hydriding reaction of LaMg_2_Ni^11–13^. In our studies, we believe that the La–H interaction formed in LaMg_2_NiH_4.5_ may drive the formation of La–H hydride (such as LaH_3_), and therefore effectively catalyze the fully hydrogenation reaction to LaMg_2_NiH_7_.

**Table 4:** The average bond order (BO), average bond length (BL, in unit of Å) and scaled bond order (BO^s^, in unit of Å^−1^) between Mg–Ni, Ni–H, La–H and Mg–H for LaMg_2_Ni, LaMg_2_NiH_4.5_ and LaMg_2_NiH_7_ and corresponding Co- and Pd-containing compounds LaMg_2_Ni–Co, LaMg_2_NiH_4.5_–Co, LaMg_2_NiH_7_–Co, LaMg_2_Ni–Pd, LaMg_2_NiH_4.5_–Pd and LaMg_2_NiH_7_–Pd according to Mulliken population analysis.

Systems	Mg–Ni			Ni–H			La–H			Mg–H		
	BO	BL	BO^s^	BO	BL	BO^s^	BO	BL	BO^s^	BO	BL	BO^s^
LaMg_2_Ni	0.475	2.775	0.171	…	…	…	…	…	…	…	…	…
LaMg_2_NiH_4.5_	0.283	2.715	0.104	0.662	1.582	0.418	0.142	2.475	0.057	0.02	2.008	0.01
LaMg_2_NiH_7_	0.117	2.723	0.043	0.741	1.581	0.469	0.163	2.485	0.066	0.041	2.379	0.017
LaMg_2_Ni–Co	0.441	2.795	0.158	…	…	…	…	…	…	…	…	…
LaMg_2_NiH_4.5_–Co	0.240	2.718	0.088	0.676	1.573	0.430	0.145	2.506	0.058	0.015	2.080	0.007
LaMg_2_NiH_7_–Co	0.110	2.722	0.040	0.724	1.619	0.447	0.159	2.485	0.064	0.045	2.275	0.020
LaMg_2_Ni–Pd	0.476	2.794	0.170	…	…	…	…	…	…	…	…	…
LaMg_2_NiH_4.5_–Pd	0.279	2.719	0.103	0.663	1.580	0.420	0.152	2.475	0.061	0.022	2.120	0.010
LaMg_2_NiH_7_–Pd	0.126	2.724	0.046	0.740	1.582	0.468	0.173	2.486	0.070	0.049	2.335	0.021

**Figure 5:**
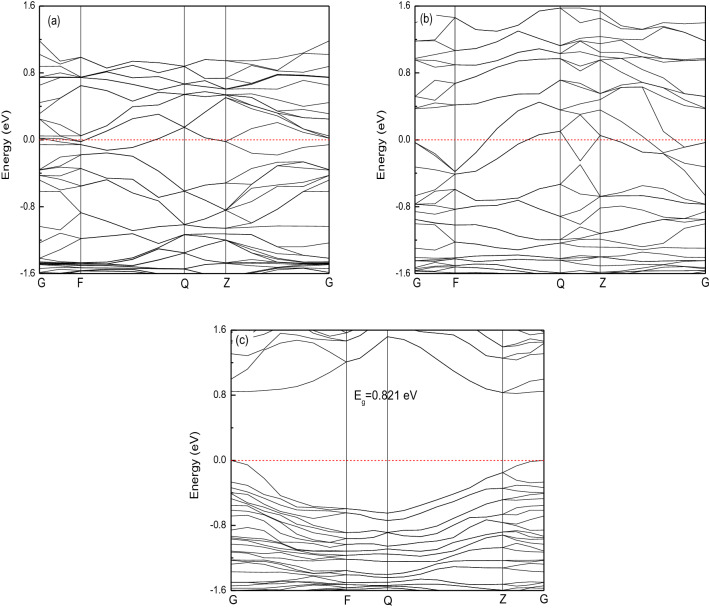
The band structure for studied compounds, (**a**) LaMg_2_Ni, (**b**) LaMg_2_NiH_4.5_, (**c**) LaMg_2_NiH_7_.

Figure 5 presents the band structure of bulk LaMg_2_Ni, LaMg_2_NiH_4.5_ and LaMg_2_NiH_7_, where the Fermi level is set at zero energy; the band gap (E_g_) characterized as the gap between the lowest energy of conduction band and the highest energy of valence band is shown in the inset of this figure. Obviously, for the host compound LaMg_2_Ni, the valence and conduction bands overlap considerably and there is no band gap at the Fermi level, as illustrated in Fig. 5a. As a result, LaMg_2_Ni will show metallic property, which is consistent with the experimental report^16^. The characteristic of band structure for LaMg_2_Ni is also reflected on the intermediate hydride LaMg_2_NiH_4.5_, i.e., LaMg_2_NiH_4.5_ also has metallic nature (Fig. 5b). For the full hydride LaMg_2_NiH_7_, the band gap is predicted to be 0.821 eV using GGA method. This value is close to the literature finding of 0.9 eV (GGA value)^16^, but is expected to be underestimated due to the strong on-site Coulomb interactions at d and f electronic states^41–43^. In general, a good agreement for band gap between theory and experiment can be obtained by adjusting the Hubbard U using GGA + U calculations^41–43^. In the present study, GGA + U calculation with different Hubbard U for La-5*d* and Ni-3*d* electrons has been employed on LaMg_2_NiH_7_, and found that the band gap of 1.454 eV at U = 3 eV for La and U = 6 eV for Ni is in the prediction by Yvon et al.^16^. Here, whatever LaMg_2_NiH_7_ has the band gap E_g_ = 0.821 eV (GGA value) or E_g_ = 1.454 eV (GGA + U value), this compound is expected to have insulator nature, and the result agrees well with the experimental finding^16^. In general, a high energy barrier associated with metal–insulator transition is expected during complex hydrogenation reaction from host metals to hydride nonmetals, because the local charge neutrality condition for complex hydrides becomes a strong constraint^17^. In our studies, because the host compound LaMg_2_Ni and the intermediate hydride LaMg_2_NiH_4.5_ are both metallic, the dehydrogenation reaction between them may be free from the energy barrier associated with the metal–insulator transition. In fact, the hydrogenation reaction of LaMg_2_Ni to LaMg_2_NiH_4.5_ process even at room temperature^17,19^. Once LaMg_2_Ni is hydrogenated to form LaMg_2_NiH_4.5_, this metallic intermediate hydride LaMg_2_NiH_4.5_ with Ni–H covalent bonds may act as precursor state for the following complex nonmetallic hydride LaMg_2_NiH_7_ formation (as described above). This will help to reduce the energy barrier for the hydrogenation reaction of LaMg_2_Ni to LaMg_2_NiH_7_ via intermediate phase LaMg_2_NiH_4.5_^17^. As in experiment, the hydrogenation reaction of LaMg_2_Ni to LaMg_2_NiH_7_ proceeds under moderate conditions (< 200 ℃, < 0.8 MPa)^15^. A similar example can be found in YMn_2_-H system. YMn_2_ reacts with hydrogen to form YMn_2_H_6_ via metallic interstitial hydride YMn_2_H_4.5_ under relatively moderate conditions, at 423 K and 5 MPa H_2_^44^.

### Hydrogen adsorption on surface

As described above, the formation of La–H bond and the suppression of Ni–H interactions are believed to favor for LaMg_2_Ni–H formation. To further understand the impact of Ni–H and La–H on LaMg_2_Ni hydrogenation, hydrogen adsorption on LaMg_2_Ni (100) surface is investigated, with the initial positions of H on the bridge site of La–Ni atoms, the top site of La atom and the top site of Ni atom (Fig. 2). The hydrogen adsorption energy (E_ads_) on the surface is expressed as following:

5 $$ E_{ads} = E_{sur(100)/H} - E_{sur(100)} - E_{H} $$

where E_sur(100)/H_ is the total energy of H-adsorbed systems, E_sur(100)_ is the total energy of H-free systems, and E_H_ is the total energy of adsorbate H. E_H_ is estimated to be − 15.895 eV by the energy of H_2_ (− 31.79 eV, as described above). The La-H and Ni–H bonding characteristics, including the bond order (BO), bond length (BL) and scaled bond order (BO^s^), are studied by Mulliken population analysis. Table 2 lists the hydrogen adsorption energies (E_ads_), as well as the bond order, the bond length, and the scaled bond order between La–H and Ni–H. As seen in Table 2, a presence of La–H and Ni–H bonds can be detected on all H-adsorbed LaMg_2_Ni (100) systems, except for an absence of Ni–H bond on the system with the initial H on the top site of La atom. And this absence may be ascribed to the long initial Ni–H distance (> 3.536 Å, Fig. 2b). H adsorption, on the one hand, leads the La–H distance to be close to that in LaH_3_ hydride (2.43 Å^16^), especially for initial H adsorption on the top site of La atom (BL_La–H_ = 2.437 Å), suggesting the intake of H may drive the formation of La–H hydride upon LaMg_2_Ni hydrogenation. On the other hand, H adsorption results a shorter Ni–H distance due to Ni is attractive to H, as compared to its initial distance. The hydrogen adsorption energy E_ads_ is calculated to be − 0.565 eV (La-Ni bridge site), − 0.433 eV (Top site of La) and − 0.311 eV (Top site of Ni). In general, a negative E_ads_ is expected to be an exothermic reaction, and H atoms can adsorb on the surface stably. Moreover, an H atom with lower adsorption energy is easier to be adsorbed on the surfaces. Obviously, H atom considered here prefers to adsorb on the bridge site of La–Ni atoms to form La–H and Ni–H bonds simultaneously. Furthermore, the formed Ni–H bond interaction with BO^s^_Ni–H_ = 0.378 Å^−1^ is stronger than the formed La–H bond interaction with BO^s^_La–H_ = -0.019 Å^−1^. This suggests Ni atom is an active site on La–Mg–Ni alloy surface for H adsorption. Similar result is found on La–Ni systems^25,26^. It is worth noting that a H-adsorbed LaMg_2_Ni (100) system with relatively lower hydrogen absorption energy exhibits rather weaker Ni–H interactions, as noted E_ads_ = − 0.565 eV and BO^s^_Ni–H_ = 0.378 Å^−1^ for La–Ni bridge site system vs. E_ads_ = − 0.311 eV and BO^s^_Ni–H_ = 0.480 Å^−1^ for top site of Ni system in Table 2. We believe that the hydrogenation ability of LaMg_2_Ni should be improved if the Ni–H interactions are suppressed.

## Conclusions

Electronic structures of LaMg_2_Ni and its hydrides (intermediate phase LaMg_2_NiH_4.5_ and fully hydrogenated phase LaMg_2_NiH_7_) were systematically investigated using first-principles density functional theory calculations, in comparison with those of corresponding Co- and Pd-doped compounds (LaMg_2_Ni–Co, LaMg_2_NiH_4.5_–Co, LaMg_2_NiH_7_–Co, LaMg_2_Ni–Pd, LaMg_2_NiH_4.5_–Pd and LaMg_2_NiH_7_–Pd). Hydrogenation behavior on LaMg_2_Ni (100) surface was also studied. Our studies aim at providing new insights into the hyrogenation of LaMg_2_Ni. The results show the hydrogenation of LaMg_2_Ni to full hydride LaMg_2_NiH_7_ is energetically favorable, as the metallic intermediate hydride LaMg_2_NiH_4.5_ with Ni–H covalent bonds may act as the precursor state for LaMg_2_NiH_7_ formation. The suppression of Mg–Ni and Ni–H interactions coupled with the formation of La–H bond may improve the hydrogenation performance of LaMg_2_Ni.
